# Innovative Treatments for Rare Anemias

**DOI:** 10.1097/HS9.0000000000000576

**Published:** 2021-06-01

**Authors:** Maria Domenica Cappellini, Alessia Marcon, Bruno Fattizzo, Irene Motta

**Affiliations:** 1 General Medicine Unit, Fondazione IRCCS Ca’ Granda Ospedale Maggiore Policlinico, Milan, Italy; 2 Department of Clinical Sciences and Community Health, Università degli Studi di Milan, Italy; 3 Hematology Unit, Fondazione IRCCS Ca’ Granda Ospedale Maggiore Policlinico, Milan, Italy; 4 Department of Oncology and Oncohematology, University of Milan, Italy

## Abstract

Rare anemias (RA) are mostly hereditary disorders with low prevalence and a broad spectrum of clinical severity, affecting different stages of erythropoiesis or red blood cell components. RA often remains underdiagnosed or misdiagnosed, and treatment options have been limited to supportive care for many years. During the last decades, the elucidation of the molecular mechanisms underlying several RA paved the way for developing new treatments. Innovative treatments other than supportive care and allogeneic bone marrow transplantation are currently in clinical trials for β-thalassemias, sickle cell disease (SCD), and congenital hemolytic anemias. Recently, luspatercept, an activin receptor ligand trap targeting ineffective erythropoiesis, has been approved as the first pharmacological treatment for transfusion-dependent β-thalassemia. L-glutamine, voxelotor, and crizanlizumab are new drugs approved SCD, targeting different steps of the complex pathophysiological mechanism. Gene therapy represents an innovative and encouraging strategy currently under evaluation in several RA and recently approved for β-thalassemia. Moreover, the advent of gene-editing technologies represents an additional option, mainly focused on correcting the defective gene or editing the expression of genes that regulate fetal hemoglobin synthesis. In this review, we aim to update the status of innovative treatments and the ongoing trials and discuss RA treatments’ future directions. Interestingly, several molecules that showed promising results for treating one of these disorders are now under evaluation in the others. In the near future, the management of RA will probably consist of polypharmacotherapy tailored to patients’ characteristics.

## Introduction

**Figure 1.**
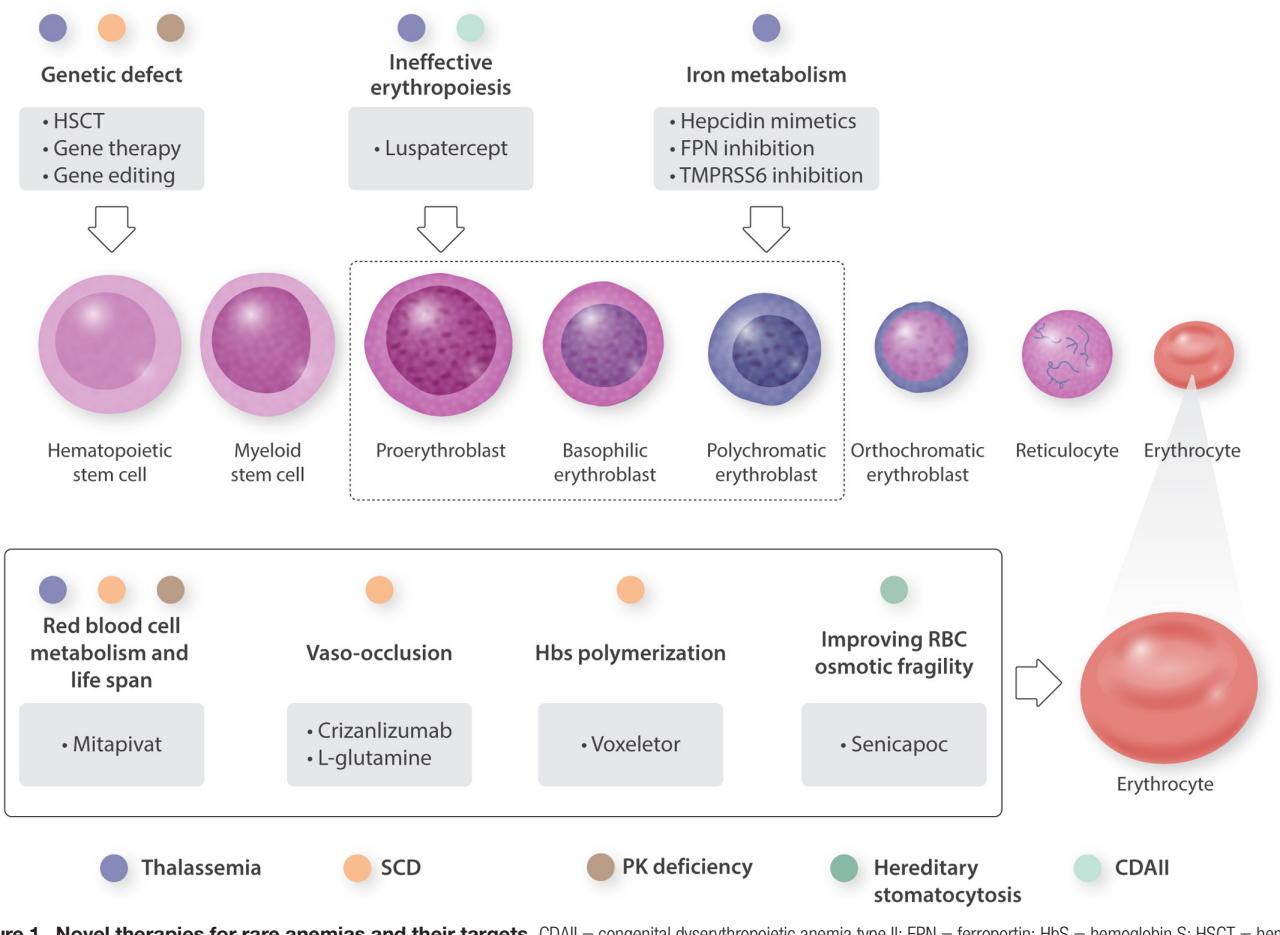
**Novel therapies for rare anemias and their targets.** CDAII = congenital dyserythropoietic anemia type II; FPN = ferroportin; HbS = hemoglobin S; HSCT = hematopoietic stem cell transplantation; RBC = red blood cell; SCD = sickle cell disease; TMPRSS6 = transmembrane serine protease 6.

Anemia is one of the most common disorders worldwide,^1^ with different underlying causes, either acquired or congenital. Rare anemias (RA) are mostly hereditary disorders with low prevalence, caused by defects that affect erythropoiesis at different stages or one of the red blood cell (RBC) components. They include Diamond-Blackfan anemia, Fanconi anemia, congenital dyserythropoietic anemias (CDAs), thalassemias, sickle cell disease (SCD), RBC enzyme deficiencies, and membrane disorders. RA also include conditions due to altered iron metabolism, as iron-refractory iron deficiency anemia.^2^ Given their rarity, RA remain often underdiagnosed or misdiagnosed, and treatment options had been limited to supportive care for many years. However, during the last decade, thanks to the improvement in pathophysiology understanding and new molecular technologies, the diagnostic ability has improved, allowing the identification of the genetic defect. Moreover, the elucidation of molecular mechanisms paved the way for the development of new treatments, including recently approved options (Figure 1), which have the potential to change the natural history of these disorders. We herein focus on the most common forms of RA, namely thalassemias, SCD, and congenital hemolytic anemias (CHAs), and their innovative treatment options, which are under evaluation in clinical trials or have been recently approved. Interestingly, promising molecules initially tested in one of these disorders, have since been investigated for other RA exploiting shared features and pathophysiology overlap, thus extending the spectrum of potential therapeutic options.

## Pharmacological therapies

### β-thalassemias

Conventional management of β-thalassemias includes RBC transfusion for the most clinically severe forms (transfusion-dependent thalassemia [TDT]), iron chelation therapy, and in selected cases, splenectomy for both TDT and non-transfusion dependent thalassemia (NTDT).^3,4^ In the last few decades, there have been considerable advances in optimizing transfusion programs and iron-chelation therapy; however, there are many challenges and limitations in the currently available conventional strategies. Emerging therapies aim to correct ineffective erythropoiesis, iron overload and dysregulation (Table 1), and genetic defect (Table 4; Figure 1).^5^

**Table 1:** New Pharmacological Treatments Under Investigation or Recently Approved for β-thalassemia.

Drug	Sponsor	NCT	Mechanism (Target)	Administration	Age	Clinical Phase/Status
Approved						
Luspatercept (TDT)	BMS/Celgene	02604433	Ineffective erythropoiesis	Subcutaneous every 21 d	≥18 y	FDA approved on November 2019; EMA approved on July 2020
Ongoing						
Sotatercept (TDT and NTDT)	BMS/Celgene	01571635	Ineffective erythropoiesis	Subcutaneous every 21 d	≥18 y	Phase 2: not recruiting
Luspatercept (NTDT)	BMS/Celgene	03342404	Ineffective erythropoiesis	Subcutaneous every 21 d	≥18 y	Phase 2: not recruiting
Luspatercept (TDT and NTDT)	BMS/Celgene	04064060	Ineffective erythropoiesis	Subcutaneous every 21 d	≥18 y	Phase 3: recruiting
Luspatercept (TDT)	BMS/Celgene	04143724	Ineffective erythropoiesis	Subcutaneous every 21 d	6 mo to 18 y	Phase 2: not yet recruiting
Ruxolitinib (TDT)	Novartis Pharmaceuticals	02049450	Ineffective erythropoiesis	Oral BID	≥18 y	Phase 2: completed
Mitapivat (TDT)	Agios Pharmaceuticals, Inc.	04770779	Ineffective erythropoiesis	Oral BID	≥18 y	Phase 3: recruiting
Mitapivat (NTDT)	Agios Pharmaceuticals, Inc.	04770753	Ineffective erythropoiesis	Oral BID	≥18 y	Phase 3: recruiting
VIT-2763 (NTDT)	Vifor (International) Inc.	04364269	Iron metabolism	Oral QD or BID	12–65 y	Phase 2: recruiting
IONIS TMPRSS6-LRx (NTDT)	Ionis Pharmaceuticals, Inc.	04059406	Iron metabolism	Subcutaneous every 4 wk	18–65 y	Phase 2: recruiting
PTG-300, (TDT and NTDT)	Protagonist Therapeutics, Inc.	03802201	Iron metabolism	Subcutaneous every wk/every 2 wk/twice a wk	12–65 y	Phase 2: stopped
LJPC-401	La Jolla Pharmaceutical Company	03381833	Iron metabolism	Subcutaneous every week	≥18 y	Phase 2: stopped

BID = twice per day; BMS = Bristol Myers Squibb; EMA = European Medicine Agency; FDA = Food and Drug Administration; NCT = National Clinical Trial number; NTDT = non-transfusion dependent thalassemia; QD = daily; TDT = transfusion dependent thalassemia.

**Table 4:** Gene Therapy and Gene Editing Trial.

Drug Product (Strategy)	Disease	Sponsor	NCT Number	Strategy	Conditioning	Age	Clinical Phase/Status
Approved							
HLA-matched transplant	TDT	NA	NA	Allogenic HSCT	Myeloablative	Pediatric and young adults^*a*^	Standard of care
	SCD						
betibeglogene autotemcel (Gene therapy)	TDT	bluebird bio	02906202	IV infusion of autologous CD34+ transduced with LentiGlobin BB305 LV encoding the β-A-T87Q-globin gene	Myeloablative (busulfan)	>12 y	EMA approved 2019
			03207009				
			02151526				
Ongoing							
LentiGlobin BB305 (Gene therapy)	SCD	Bluebird bio	04293185	IV infusion of autologous CD34+ transduced with LV encoding the β-A-T87Q-globin gene	Myeloablative (busulfan)	2–50 y	Phase 3: recruiting
			02151526				
GLOBE^66^	TDT	TIGET	02453477	Intrabone autologous CD34+ transduced with the GLOBE LV encoding for the β-globin gene	Myeloablative (treosulfan and thiotepa)	3–64 y	Phase 1/2: active, not recruiting
ST-400 (Gene editing)	TDT	Sangamo Therapeutics	03432364	IV infusion of autologous CD34+ modified ex vivo at the erythroid-specific enhancer of the BCL11A gene (ZNF)	Myeloablative (busulfan)	18–40 y	Phase 1/2: active, not recruiting
BCH-BB694 (Gene therapy)	SCD	Boston Children Hospital	03282656	IV infusion of autologous bone marrow derived CD34+ transduced with the LV containing a shRNA targeting BCL11A	Myeloablative (busulfan)	3–40 y	Phase 1: suspended
ARU-1801 (Gene therapy)	SCD	Aruvant Sciences GmbH	02186418	CD34+ cells transduced ex-vivo with the γ-globin LV	Reduced intensity conditioning with single-dose melphalan	18–45 y	Phase 1/2: active, not recruiting
DREPAGLOBE (Gene therapy)	SCD	Assistance Publique - Hôpitaux de Paris	03964792	Autologous CD34+ transduced ex vivo with the GLOBE1 LV expressing the βAS3 globin gene	Not known	5–35 y	Phase 1/2: recruiting
Lenti/βAS3-FB (Gene therapy)	SCD	University of California	02247843	CD34+ from the peripheral blood transduced ex-vivo with the Lenti/βAS3-FB LV	Busulfan	≥18 y	Phase 1/2: recruiting
CSL200 (Gene therapy)	SCD	CSL Behring	04091737	Autologous enriched CD34+ cell fraction that contains CD34+ cells transduced with LV encoding human γ-globinG16D and shRNA734	Reduced intensity conditioning with melphalan	18–45 y	Phase 1: active, not recruiting
RP-L301 (Gene therapy)	PKD	Rocket Pharmaceuticals Inc.	04105166	IV infusion of autologous CD34+ transduced with PK gene	Not known	8–50 y	Phase 1: recruiting
CTX001 (Gene editing)	TDT	Vertex Pharmaceuticals Inc.	03655678	IV infusion of autologous CD34+ modified with at the erythroid lineage-specific enhancer of the BCL11A gene (CRISPR-Cas9)	Myeloablative (busulfan)	12–35 y	Phase 1/2: recruiting
	SCD	CRISPR Therapeutics	03745287				
OTQ923 and HIX76 (Gene editing)	SCD	Novartis Pharmaceuticals	04443907	Autologous, HSPC products—OTQ923 and HIX763—each reducing the biologic activity of BCL11A	Not known	2–40 y	Phase 1/2: recruiting
BIVV003 (Gene editing)	SCD	Bioverativ, a Sanofi company	03653247	IV infusion of autologous CD34+ transfected ex vivo with ZFN mRNAs targeting the BCL11A locus	Myeloablative (busulfan)	18–40 y	Phase 1/2: recruiting

^*a*^Pesaro criteria for patient selection are recommended.^53^

BCL11A = B-cell lymphoma/leukemia 11A; CRISPR = clustered regularly interspaced short palindromic repeats; EMA = European Medicine Agency; HLA = human leukocyte antigen; HSCT = hematopoietic stem cell transplantation; HSPC = hematopoietic stem and progenitor cell; LV = lentiviral vector; NA = not applicable; NCT = National clinical trial; PK = pyruvate kinase; PKD = pyruvate kinase deficiency; SCD = sickle cell disease; shRNA = short hairpin RNA; TDT = transfusion dependent thalassemia; TIGET = Telethon Institute for Gene Therapy; ZNF = zinc finger.

Improvement in ineffective erythropoiesis, the hallmark of the disease, is one of the main therapeutic goals in thalassemia syndromes. Over the last years, two promising twin molecules (sotatercept and luspatercept) have been studied in this field. They act as activin receptor (ACVR) ligand traps by binding the transforming growth factor (TGF)-β–like molecules through the extracellular domain of the ACVR type 2A (ACVR2A) or 2B (ACVR2B). Ligand scavenging prevents the activation of the downstream signaling pathways^6^ and modulates the differentiation of late-stage erythrocyte precursors in the bone marrow leading to improved erythropoiesis and a consequent increase of Hb values.^7^ Even if sotatercept showed promising results in clinical trials,^8^ luspatercept was selected for phase 3 development, mainly because it binds activin B, growth differentiation factor (GDF)8, and GDF11, but not with activin A, and consequently shows a higher ligand selectivity making this specific molecule more suitable for treating anemia and ineffective erythropoiesis with lower off-target effects.^9^ In the BELIEVE trial (NCT 02604433)—a phase 3 multicenter international randomized, double-blind, placebo-controlled trial—336 adult patients with TDT were enrolled and randomized in a 2:1 ratio to receive luspatercept subcutaneously plus best supportive care (BSC) versus placebo plus BSC every 3 weeks for at least 48 weeks. The primary endpoint was the percentage of patients with a transfusion burden reduction of at least 33% from baseline (12 wk before the first dose of luspatercept or placebo) during weeks 13 through 24, plus a reduction of at least 2 red-cell units over this 12-week interval. Other efficacy endpoints included reductions in the transfusion burden during any 12-week interval, and iron status results. Forty-eight out of 224 (21.4%) patients in the luspatercept group achieved the primary endpoints compared to the placebo group (4.5%; *P* < 0.001). Moreover, during any 12-week interval, the percentage of patients who had a reduction in transfusion burden of at least 33% was greater in the luspatercept group than in the placebo group (70.5% vs 29.5%). Also, the percentage of patients who had a reduction of at least 50% was significantly higher in the luspatercept group (40.2% vs 6.3%). Transfusion independence was achieved by 11% of the patients in the luspatercept group during any 8-week interval. Adverse events (AEs) of transient bone pain, arthralgia, dizziness, hypertension, and hyperuricemia were more frequent with luspatercept than placebo, but mild and manageable. An initial decrease of ferritin level at week 48 in the luspatercept group was also reported. This reduction may be attributed either to a reduced iron intake, or, possibly to an improved iron utilization (more effective erythropoiesis and red cell production).^10^ Following this trial’s results, the US Food and Drug Administration (FDA) in 2019^11^ and the European Medicines Agency (EMA) in 2020^12^ approved luspatercept (Reblozyl; Bristol Myers Squibb/Celgene Corp.) for treatment of anemia in adult patients with β-thalassemia who require regular RBC transfusions. The recommended starting dose of luspatercept is 1 mg/kg once every 3 weeks by subcutaneous injection. No contraindications are reported in both FDA and EMA product information descriptions, except for hypersensitivity to the active substance or any of the excipients and pregnancy. The potential benefit of treatment with luspatercept should be weighed against the risk of thromboembolic events in splenectomized β-thalassemia patients and with other risk factors (eg, history of thrombocytosis or concomitant use of hormone replacement therapy). Although benefit was observed in all subgroups, genotype analyses suggest that the response is lower in patients with a β0/β0 genotype compared to those with a non-β0/β0 genotype.^13^ However, we expect that in real-life use, most TDT patients could benefit from this drug to reduce transfusion burden and/or increase pre-transfusional hemoglobin (Hb) values, thus ameliorating their quality of life. A phase 2 trial (BEYOND NCT 03342404) in patients with NTDT is ongoing, and core data will be presented at the European Hematology Association 2021 meeting; a pediatric study is ready to start. Moreover, in a phase 3 clinical trial (MEDALIST, NCT02631070), luspatercept has demonstrated to significantly reduce the transfusion burden in a substantial proportion of patients with lower-risk myelodysplastic syndromes (MDS) with ring sideroblasts.^14^ The ongoing COMMANDS trial (NCT03682536) is evaluating the efficacy of luspatercept in other subgroups of patients with MDS.

Several pre-clinical studies have provided evidence on the role of Janus kinase 2 (JAK2) as a potential target to treat disorders with ineffective erythropoiesis.^15^ The inhibition of JAK2 in TDT and NTDT mouse models improved ineffective erythropoiesis and reversed splenomegaly.^16^ A phase 2a study (NCT02049450) assessed ruxolitinib efficacy and safety in TDT patients with spleen enlargement. A decrease in spleen size from baseline was observed in ruxolitinib-treated patients. However, no clinically significant improvements in pre-transfusion Hb have been observed, with no reduction in transfusion needs. For these reasons, the study did not proceed into phase 3.^17^ Ineffective erythropoiesis can also be targeted through a different approach generating iron-restricted erythropoiesis.^18^ This can be achieved by acting on iron metabolism in thalassemias, especially on hepcidin that is the master regulator of iron metabolism. Iron-restricted erythropoiesis is associated with a reduction in the formation of hemichromes and an improved lifespan of RBCs.^19–21^ However, the exact mechanism through which iron restriction ameliorates erythropoiesis has not yet been elucidated. A substantial number of agents that mimic hepcidin activity or stimulate its expression have been investigated in pre-clinical phase, demonstrating a beneficial activity. While clinical trials with hepcidin mimetics have been prematurely terminated due to the absence of efficacy (molecule La Jolla Pharmaceutical Company-401, NCT03381833; molecule PTG-300, TRANSCEND trial, NCT03802201), it remains to be determined whether molecules regulating hepcidin expression or ferroportin function are more effective at improving anemia in thalassemia patients.

A more recent approach targets ferroportin. VIT-2763 is a small oral molecule that acts as ferroportin inhibitor. The promising pre-clinical^22,23^ and phase 1^24^ studies have led to a phase 2 trial by Vifor Pharma that is currently recruiting NTDT patients, assessing the efficacy, safety, tolerability, pharmacokinetics, and pharmacodynamics of VIT-2763 in this patient population (NCT04364269).

Mouse and human genetic data indicated that lowering transmembrane serine protease 6 (TMPRSS6) expression could up-regulate hepcidin and ameliorate many of the disease symptoms associated with beta-thalassemia.^25–27^ A phase 2a, randomized, open-label study to evaluate the efficacy, safety, tolerability, pharmacokinetics and pharmacodynamics of ISIS 702843 administered subcutaneously to patients with NTDT is ongoing (Ionis Pharmaceuticals, Inc., NCT04059406).

Mitapivat (AG-348), a first-in-class, small-molecule, oral activator of RBC pyruvate kinase (PK)—a key enzyme regulating ATP production via glycolysis—showed promising results in PK deficiency (PKD; see Congenital Hemolytic Anemias) and in a mouse model of β-thalassemia. ATP supply appears to be insufficient in thalassemic RBCs to maintain membrane fitness and clearance of globin precipitates. PK-R is a key enzyme for maintaining energy homeostasis in RBC, as RBCs rely almost exclusively on the process of glycolysis to generate ATP.^28^ Thus, the drug is currently in clinical trial for both TDT (NCT 04770779) and NTDT (NCT 04770753) (Table 1). Data on NTDT patients have been presented at the last American Society of Hematology (ASH) 2020 meeting. Twelve out of 13 patients achieved a Hb increase from baseline ≥1.0 g/dL after a median of 3.1 weeks (range 1.4–7.1), including 4/4 with α-thalassemia. Markers of erythropoiesis and hemolysis also improved. The safety profile was consistent with previous published mitapivat studies with no serious adverse AEs or AEs leading to treatment discontinuation.^29^

Given the multiple drug options targeting different pathways and pathophysiological aspects of a complex disease, β-thalassemia represents a personalized medicine model that could be based on a multi-drug approach.

### Sickle cell disease

The FDA approved hydroxyurea (HU) for use in adults with SCD in 1998,^30^ and for 20 years it has been the only pharmacological option in these patients. Its ability to induce fetal hemoglobin (HbF) and reduce hemolysis and adhesion receptor expression, along with the capability to improve RBC deformability, makes this drug a milestone in SCD treatment. A large number of studies demonstrated that HU ameliorates SCD morbidity by decreasing the risk of vaso-occlusive crises (VOCs), acute chest syndrome (ACS), stroke, and chronic kidney disease.^31^ Because of its favorable safety profile and efficacy, HU is recommended for people of all ages with SCD.^32^ However, HU continues to be underutilized due to a combination of provider- and patient-related factors. The principal reason is patients’ low compliance due to HU potential side effects (gastrointestinal toxicity, such as nausea and anorexia), the need for contraception, and monitoring laboratory tests for potential myelosuppression. Moreover, some physicians hesitate to prescribe HU because of concerns over its carcinogenicity, effectiveness, and teratogenicity.^33^ Also, there is no clear evidence of HU long-term effect in preventing end-organ damage, and its effects on male fertility are still not elucidated.^34,35^ For these reasons, new therapeutic approaches for SCD targeting the complex pathophysiological mechanisms have been developed and are under evaluation (Figure 1).

Following a large number of clinical trials (Table 2) over the last 5 years, the FDA has approved 3 new drugs for SCD treatment. L-glutamine is the first among the recently approved drugs for adults and children (>5 years old).^36^ Oral administration of L-glutamine raises the nicotinamide adenine dinucleotide redox ratio within sickle cells and is associated with patient-reported clinical improvement.^37^ In a phase III trial (NCT01179217) a reduction in pain crisis in both children and adults treated with L-glutamine, alone or with HU, was demonstrated.^38^ However, L-glutamine clinical benefit appears to be modest, and L-glutamine supplementation should be used cautiously in SCD patients with hepatic or renal impairment.^39^

**Table 2:** New Pharmacological Treatments Under Investigation or Recently Approved for Sickle Cell Disease.

Drug	Sponsor	NCT Number	Mechanism (Target)	Administration	Age	Clinical Phase/Status
Approved						
Hydroxyurea	NA	NA	HbS polymerization	Oral QD	>2 y	Standard of care
L-glutamine	Emmaus Medical, Inc.	01179217	Vaso-occlusion	Oral	>5 y	FDA approved in 2017
Voxelotor^*a*^	Global Blood Therapeutics	03943615	HbS polymerization	Oral	>12 y	FDA approved in 2019
Crizanlizumab^*a*^	Novartis Pharmaceuticals	03264989	Vaso-occlusion	IV infusion every 2 wk for first month, then every 4 wk	>16 y	FDA approved in 2019; EMA in 2020
Ongoing/suspended						
PDE9 inhibitor (IMR-687)	Imara, Inc.	04053803	HbS polymerization	Oral QD	≥18 y	Phase 2: enrolling by invitation
Panobinostat	Abdullah Kutlar	01245179	HbS polymerization	Oral TW	≥18 y	Phase 1: active, not recruiting
Decitabine	University of Illinois at Chicago	01685515	HbS polymerization	Oral BID on 2 consecutive days over 8 wk	≥18 y	Phase 1: recruitment completed
Memantine	HaEmek Medical Center, Israel	03247218	HbS polymerization	Oral QD	>10 y	Phase 2: active, recruiting
Sanguinate	Prolong Pharmaceuticals	02411708	HbS polymerization	Single 2-h infusion	≥18 y	Phase 1/2: recruitment completed
Mitapivat	NHLBI	04610866	HbS polymerization	Oral BID	18–70 y	Phase 1/2: recruiting
Sevuparin	Modus Therapeutics	02515838	Vaso-occlusion	2–7 d continuous IV administration	12–50 y	Phase 2: recruitment completed
Simvastatin	University of Campinas, Brazil	03599609	Inflammation	Oral QD	>35 y	Phase 1: active, not recruiting
N-acetylcisteine	University of Washington/Bloodworks Northwest	01800526	Inflammation	Oral and intravenous administration	≥18 y	Phase 1/2: completed
Rivaroxaban	University of North Carolina, Chapel Hill	02072668	Inflammation	Oral QD	18–65 y	Phase 2: completed
IVIG	Albert Einstein College of Medicine	01757418	Inflammation	Single dose of IVIG within 24 h of hospitalization	12–65 y	Phase 1/2: recruiting
Ticagrerol	AstraZeneca	02482298	Inflammation	Oral BID	18–30 y	Phase 2: completed

^*a*^Other ongoing trials.

BID = twice per day; EMA = European Medicine Agency; FDA = Food and Drug Administration; HbS = hemoglobin S; NA = not applicable; NCT = National Clinical Trial number; NHLBI = National Heart, Lung, and Blood Institute; QD = daily; TW = twice a week.

More recently, 2 other drugs, voxelotor and crizanlizumab, were approved by FDA and EMA. Voxelotor is a Hb modulator that increases oxygen affinity with a consequent polymerization inhibition.^40^ In a phase 3 trial (NCT03036813) it was demonstrated that oral administration of voxelotor significantly increased Hb levels and reduced hemolysis markers due to the inhibition of hemoglobin S (HbS) polymerization.^41^ Voxelotor was tested in patients >12 years old, and its effect was demonstrated to be dose-dependent. Moreover, the reduction in hemolysis was demonstrated to be independent of HU, and the increase in Hb levels was not associated with an increased number in VOCs.^42^ Despite the increase in Hb levels and the reduction in hemolysis, the VOC reduction was non-significant.^41^ For this reason, voxelotor may represent a therapeutic option in patients with hemolytic complications, such as severe anemia, more than in patients with frequent and severe VOCs.

Crizanlizumab was approved by FDA in 2019 and by EMA in 2020 for the treatment of SCD patients older than 16 years.^43^ Crizanlizumab is a monoclonal antibody that binds P-selectin, the principal mediator of vaso-occlusion, and, consequently, blocks activated erythrocytes neutrophil and platelet adhesion.^44^ The phase 2, multicenter, double-blind, randomized, placebo-controlled clinical trial (NCT01895361) that led to the drug approval demonstrated a significant reduction in vaso-occlusion rate and a longer median time to the first VOC.^44^ The clinical benefits were observed regardless of HU use. A careful pharmacovigilance program must be implemented due to the potential infection risk related to the P-selectin block.^45^

Several other molecules are under evaluation targeting different pathophysiological mechanisms such as inflammation, adhesion, and oxidative stress.^46^ It is possible that in the near future, the optimization of SCD treatment will require a multi-drug approach using different molecules that target different steps in the SCD pathological pathway and is tailored to the phenotype and clinical manifestation of the patient. However, the combination of different drugs may impact patients’ compliance and health systems sustainability.

### Congenital hemolytic anemias

CHAs are heterogeneous conditions, with either dominant, recessive, or X-linked inheritance, displaying a clinical course ranging from mild, fully compensated anemia to chronic severe hemolysis requiring transfusion support. For symptomatic, severely anemic patients, transfusions (along with vitamins support and iron chelation) and splenectomy (combined with cholecystectomy in case of gallstones) have been the only therapeutic approaches for decades. However, while splenectomy leads to substantial anemia recovery in hereditary spherocytosis (HS), it is considerably less effective in PKD and CDAs, and most patients remain transfusion-dependent (median Hb increase of 3 g/dL in HS, 1.6–1.8 g/dL in PKD, and 1 g/dL in CDA type II). Moreover, sepsis and thrombotic events have been reported, particularly in PKD with a frequency of ~7% for both and in hereditary stomatocytosis (HSt) where splenectomy is contraindicated. Novel therapeutic strategies are being actively explored in CHAs, including erythrocyte membrane defects (ie, HS, elliptocytosis, and HSt), enzymopathies (ie, PKD, glucose-phosphate isomerase deficiency, etc), and the very rare CDAs (Table 3). The most promising and in advanced stage of study is mitapivat (formerly AG348), an oral, small-molecule allosteric activator of PK in red cells. Mitapivat safety and efficacy has been evaluated in a phase 2 global trial (NCT02476916) enrolling 52 adults with non-transfusion dependent PKD. Fifty percent of patients showed a Hb increase (>1 g/dL), and the response persisted after a median follow-up of 29 months (range 22–35). Importantly, Hb responses were observed only in patients with at least 1 missense *PKLR* mutation and were associated with the red-cell PK protein level at baseline.^47^ Based on these promising data, 2 further phase 3 trials in transfusion-dependent and non-transfusion–dependent PKD adult patients are currently ongoing (NCT03559699; NCT03548220). The observation that mitapivat is not active on patients with more disruptive *PKLR* mutations and that it does not improve the disease represent additional unmet needs and stimulated further investigational approaches, such as gene therapy (discussed in a separate paragraph). Regarding membrane defects, HSt is due to mutations affecting cation channels, resulting in a shrinkage (dehydrated HSt) or swelling (overhydrated HSt) of the RBCs. Dehydrated stomatocytosis caused by a mutation in the Gardos channel, *KCNN4*, has recently received more attention. V282M mutation of the *KCNN4* gene results in a hyperactivated channel that leads to erythrocyte dehydration. Senicapoc, a selective blocker of the Gardos channel previously studied in SCD, may revert erythrocyte dehydration and recover erythrocyte lifespan. A phase 1 proof-of-concept study (NCT04372498) in patients with V282M mutated HSt is currently ongoing.

**Table 3:** New Pharmacological Treatments Under Investigation for Congenital Hemolytic Anemias.

Drug (Disease)	Sponsor	NCT Number	Mechanism (Target)	Administration	Age	Clinical Phase/Status
Mitapivat (PKD)	Agios Pharmaceuticals	02476916, 03559699, 03548220, 03853798	Allosteric PK enzyme activator	Oral 20–300 mg BID	≥18 y	Phase 2: available results; phase 3: enrolment closed
Senicapoc (dehydrated HSt)	Boston Children’s Hospital	04372498	Inhibitor of the cation Gardos channel	Oral 10–40 mg a day	≥21 y	Phase 1 and 2: not yet recruiting
Luspatercept and Sotatercept (CDAII)	Bristol-Meyer-Squibb	-	Inhibitors of TGF-β superfamily molecules	Subcutaneous 1–1.75 mg/kg every 3 wk	-	Pre-clinical evidences
	Acceleron Pharma					
Omeprazole (CDAI)	Soroka University Medical Center	01795794	Hydrogen pump inhibitor	Oral 20 mg QD	≥12 y	Phase 4

BID = twice per day; CDAI = congenital dyserythropoietic anemia type I; CDAII = congenital dyserythropoietic anemia type II; HSt = hereditary stomatocytosis; NCT = National Clinical Trial number; PKD = pyruvate kinase deficient; QD = daily; TGF = transforming growth factor.

A final mention is deserving for CDAs characterized by several mutations resulting in ineffective erythropoiesis. CDAs are classified into the 3 major types (I, II, III), and the use of next-generation sequencing improved the identification of new causative/modifier genes and polygenic conditions.^48^ A deeper knowledge of myelopoiesis molecular mechanisms paved the way to several drugs targeting later erythroid differentiation steps, such as those inhibiting TGF-β superfamily molecules (luspatercept and sotatercept). In vitro and ex vivo studies showed that sotatercept restores erythroid markers gene expression by inhibiting the phosphorylated SMAD2 pathway. Moreover, it reduces the expression of the erythroblast-derived hormone erythroferrone, suggesting a potential benefit for iron overload. In fact, the study of iron metabolism in CDAII showed that erythroferrone specifically inhibits hepcidin production, leading to liver iron overload even in non-transfusion dependent patients.^49^ No specific studies have been conducted on iron chelation in this setting. An interesting strategy, beyond deferoxamine, deferiprone, and the mostly used deferasirox, comes from the observation that iron is absorbed in acidic area. Thus, acidity-reducing drugs like hydrogen pump inhibitors can inhibit iron absorption, as also reported in hemochromatosis. A clinical trial (NCT01795794) with no results available yet evaluated the efficacy of omeprazole 20 mg day for 6 months in decreasing acidity and iron absorption in patients with CDA I with ferritin levels higher from the normal range, but not yet requiring medical therapy (400–700 μg/L).

## Gene therapy and gene editing

### β-hemoglobinopathies

Allogeneic hematopoietic stem cell (HSC) transplantation (HSCT) has been the only curative option for hemoglobinopathies for decades^50^ until June 2019, when the betibeglogene autotemcel (LentiGlobin BB305 [Zynteglo]), the first gene-addition product, was approved by EMA for TDT patients aged over 12 years who do not have a β0/β0 genotype, and for whom HSCT is appropriate but a human leukocyte antigen (HLA)-matched related HSC donor is not available.^51^ Of note, the availability of a suitable donor, patient fitness,^52,53^ and graft-versus-host disease (GVHD) remain the main limitations to HSCT. Several clinical trials are currently investigating the safety and efficacy of gene addition and gene editing in β-thalassemia and SCD (Table 4). According to different strategies, the goal is to produce exogenous β- or γ-globin chains, induce endogenous γ-globin production, or correct the SCD mutation.^54^ This will restore the α/β-like imbalance in thalassemia patients and reduce the percentage of HbS in SCD.

During the development of the approved betibeglogene autotemcel, after the first clinical trials,^55^ the sponsor—Bluebird Bio—improved the transduction process, thereby significantly increasing the vector copy number of the investigational products. According to the last data presented at the ASH 2020 meeting, 60 patients with TDT have been treated across 2 completed phase 1/2 studies (HGB-204, NCT01745120; HGB-205, NCT02151526) and in 2 ongoing phase 3 studies (HGB-207, NCT02906202; HGB-212; NCT03207009). As of March 2020, among the 32 patients enrolled in the long-term follow-up study, with a mean post-infusion follow-up of 49.1 (range 23.3–71.8) months, transfusion independence (defined as a weighted average Hb ≥9 g/dL without packed RBC transfusions for ≥12 mo) was achieved in 14 of 22 (64%) patients treated in phase 1/2 and in 9 of 10 (90%) patients treated in phase 3. This was associated with an improved quality of life.^56^

The same product is under investigation in SCD patients in a phase 1/2 study (HGB-206, NCT02140554), with 25 patients treated according to the last (2020) ASH meeting data. All patients stopped RBC transfusions by 90 days post-treatment. Complete resolution of VOC and ACS was observed in almost all patients. In addition, overall, patients reported an improved pain intensity score.^57^

Further new strategies take advantage of the observation that the naturally occurring mutations causing hereditary persistence of HbF ameliorate SCD and TDT clinical manifestations. B-cell lymphoma/leukemia 11A (BCL11A) is a transcription factor that represses γ-globin expression and has been identified as a target to restore high expression of HbF.^58^ The infusion of autologous CD34+ cells transduced with the BCH-BB694 lentiviral vector, which encodes a short hairpin RNA targeting in the erythroid lineage BCL11A mRNA embedded in a microRNA, led to a robust and stable HbF induction in 6 patients in a phase 1 study (NCT03282656), with marked clinical improvements in the majority of patients.^59^

The clustered regularly interspaced short palindromic repeats (CRISPR)-Cas9 nuclease system is another strategy used to recapitulate the phenotype of hereditary persistence of fetal hemoglobin. The drug CTX001 was prepared by genetically modifying CD34+ hematopoietic stem and progenitor cells with CRISPR-Cas9 editing to decrease BCL11A expression in erythroid-lineage cells. This was obtained by specifically targeting the erythroid-specific enhancer region of BCL11A.^60^ Updated data were presented at the last (2020) ASH meeting about 5 TDT patients (NCT03655678) and 2 SCD patients (NCT03745287). All patients demonstrated increases in total Hb and HbF over time, with all 5 patients with TDT becoming transfusion-independent approximately 2 months after CTX001 infusion, and the 2 patients with severe SCD having had no VOC during follow-up after CTX001 infusion. All patients infused with CTX001 demonstrated hematopoietic engraftment with a post-infusion safety profile generally consistent with myeloablation.^61^

The gene therapy and gene editing process carries risks and challenges,^62^ especially concerning conditioning regimens. At the time of writing this review, the phase 1/2 (HGB-206) and phase 3 (HGB-210, NCT04293185) studies of lentiviral vector BB305 gene therapy for SCD, as well as the marketing of Zynteglo, are temporary suspended due to a reported Suspected Unexpected Serious Adverse Reaction of acute myeloid leukemia (AML).^63^ A case of MDS in a SCD patient treated in the HGB-206 trial, who did not have MDS before myeloablation, was also observed.^64^ The absence of vector integration in CD34+ blasts excludes lentiviral vector-mediated oncogenesis. An even more recent press release from Bluebird bio on March 10, 2021, announced that, according to the analyses, lentiviral vector BB305 unlikely caused the AML.^65^ Also, the gene therapy trial with the BCH-BB694 lentiviral vector has been paused per Data Safety Monitoring Board pending investigation of AE occurrence in an unrelated gene therapy study involving SCD patients (NCT03282656; last accessed March 14, 2021).

As observed for other therapeutic strategies, gene therapy and genome editing—once well established in terms of efficacy and safety—could be applied to other forms of RA for which the molecular defect is known.

### Pyruvate kinase deficiency

Gene therapy approaches are also moving the first steps in PKD, and a phase 1 trial is currently ongoing (NCT04105166). Autologous HSCs from mobilized peripheral blood will be transduced ex vivo with a lentiviral vector carrying a correct copy of the PK gene and then infused intravenously back to the patient. This approach will hopefully restore PK levels/activity in PKD patients.

## Conclusions

RA represent a heterogeneous group, often challenging to diagnose and until nowadays, with limited treatment options. The new molecular technologies and pathophysiological definition have improved the diagnostic approach stimulating the scientific interest in developing new therapies. Interestingly, molecules initially designed to treat one of these disorders are now under evaluation in the others (Figure 1), thus accelerating the steps of clinical trials, especially in terms of safety. A new era just started for innovative treatments, and their use in the management of RA will most likely consist in polypharmacotherapy tailored to patients’ characteristics due to the complexity and the genotypic and phenotypic variability of the disorders.

## Acknowledgments

We thank Daniela Canali for producing the artwork.

## Disclosures

MDC reports receiving consultancy, research funding and honoraria from Novartis; research funding and consultancy from Celgene; consultancy from Vifor, research funding from La Jolla, research funding from Protagonist Therapeutics; consultancy from IONIS Pharmaceuticals, and research funding from CRISPR Therapeutics. BF reports receiving consultancy honoraria from Novartis, Amgen, and Momenta; IM reports receiving consultancy honoraria from Sanofi-Genzyme and Amicus Therapeutics. The other author has no conflicts of interest to disclose.
